# Chronic Dietary Stress Is Associated with Early Cardiac Dysfunction and Progressive MASH Accompanied by Systemic Inflammation and Fibrosis

**DOI:** 10.3390/nu18183084

**Published:** 2026-09-20

**Authors:** Saima Shakil Malik, Hua Mao, Mariam Hamoudi, Xinchun Pi, Liang Xie

**Affiliations:** Department of Medicine, Cardiovascular Research Institute, Baylor College of Medicine, Houston, TX 77030, USA; saima.shakil@bcm.edu (S.S.M.); hua.mao@bcm.edu (H.M.); mariam.hamoudi@bcm.edu (M.H.); xinchun.pi@bcm.edu (X.P.)

**Keywords:** Gubra Amylin NASH (GAN) diet, cardiac dysfunction, liver remodeling, inflammation, MASH

## Abstract

Introduction: Animal models of cardiac dysfunction and heart failure play important roles in preclinical study and drug discovery. Cardiovascular disease and metabolic dysfunction-associated steatohepatitis (MASH) are closely associated metabolic disorders that frequently coexist in individuals with obesity and metabolic syndrome. However, the mechanisms linking diet-induced metabolic stress to cardiac dysfunction and hepatic pathology remain understudied. Method: In this study, six-week-old C57Bl/6J male mice were fed standard normal chow or a Gubra Amylin NASH (GAN) diet for six months to investigate the concurrent cardiac and hepatic manifestations. Results: Our results indicated that GAN diet-fed mice developed early cardiac dysfunction characterized by reduced ejection fraction (EF) and impaired diastolic function, as indicated by increased E/A and E/E′ ratios. These cardiac changes were accompanied by worsening hepatic pathology, including elevated total cholesterol (TC) and liver injury markers such as ALT, AST and ALP. By 6 months, mice developed pronounced fibrotic remodeling and inflammation in both the heart and liver, as confirmed by histopathological and gene expression analyses. Metabolic assessments further demonstrated a decreased respiratory exchange ratio (RER) in GAN diet-fed mice, particularly during the dark cycle, suggesting enhanced lipid utilization and decreased metabolic flexibility. In addition, reduced oxygen consumption, carbon dioxide production, and locomotor activity suggested impaired energy expenditure and reduced physical activity levels. Conclusion: Collectively, our findings demonstrate that chronic GAN diet feeding induces early cardiac dysfunction accompanied by progressive MASH-like hepatic pathology, with inflammation and fibrotic remodeling developing in both the heart and liver. These findings establish GAN diet feeding as a useful model for investigating the concurrent cardiac and hepatic consequences of chronic metabolic stress.

## 1. Introduction

The ever-growing burden of cardiometabolic diseases (CMDs), a group of interrelated disorders such as obesity, type 2 diabetes mellitus, ischemic heart disease, and heart failure (HF) [1], is the leading cause of death worldwide. In 2019 alone, cardiovascular diseases accounted for approximately 18.6 million deaths, representing nearly one-third of all mortalities [2]. Heart failure is a complex clinical syndrome resulting from functional or structural impairment of ventricular filling or/and blood ejection [3]. In the growing body of metabolic research, the relationship between cardiovascular disease (CVD) and metabolic-associated steatotic liver disease (MASLD) has attracted considerable attention due to their high prevalence, shared pathophysiological mechanisms, and substantial global health influence. Dyslipidemia and hypercholesterolemia exacerbate liver disease by promoting hepatic inflammation and lipid accumulation, which in turn accelerate vascular dysfunction [4]. Obesity further contributes to the progression of both diseases through oxidative stress and systemic inflammation [5]. In addition, hypertension aggravates hepatic fibrosis and vascular injury, emphasizing a shared pathophysiological pathway [6]. Altered lipid intermediates, pro-inflammatory cytokines and hepatokines collectively promote mitochondrial dysfunction, myocardial metabolic inflexibility and extracellular matrix remodeling [7].

The American Heart Association published a scientific statement in 2022 recognizing MASLD as a risk factor for atherosclerotic cardiovascular disease (ASCVD). These guidelines presented screening and diagnostic strategies, as well as insights into pathophysiology and potential interventions [8]. However, beyond these statements, little guidance has been provided for the co-diagnosis and integrated management of heart failure and MASLD. While heart failure and MASLD are traditionally managed as separate clinical entities, their frequent coexistence highlights the need for a multidisciplinary approach to diagnosis and treatment [9].

HFD (high-fat diet) models have been widely used to explore the effects of metabolic stress on the cardiac phenotype, but their effects on cardiac structure and function, particularly at early stages of disease, have been inconsistent [10]. Prolonged high-fat feeding has been reported to induce obesity, metabolic abnormalities, cardiomyocyte hypertrophy, cardiac fibrosis, and impaired cardiac function, whereas other dietary models have produced more modest or distinct cardiac phenotypes [11,12,13]. More recently, a high-fat, carbohydrate-free diet was shown to induce cardiac metabolic and mitochondrial dysfunction, although ejection fraction remained preserved [14]. These findings suggest that the cardiac response to dietary stress may depend on the composition and duration of the diet. Importantly, HFD alone does not consistently produce metabolic dysfunction-associated steatohepatitis (MASH) [11], previously termed non-alcoholic steatohepatitis (NASH), together with measurable cardiac dysfunction in mice [12]. Thus, conventional dietary models may not fully recapitulate the complex metabolic stress required to produce progressive cardiac dysfunction while simultaneously reproducing the hepatic features of MASH.

Researchers have therefore increasingly adopted the Gubra Amylin NASH (GAN) diet, a high-fat, high-fructose, high-cholesterol diet (40 kcal% fat, 22% fructose, 2% cholesterol) that induces obesity and a pronounced MASH phenotype in mice after 28 weeks of feeding, characterized by increased steatosis and fibrosis [15,16]. Although the GAN diet has strong relevance to human metabolic liver disease and has been extensively used to investigate hepatic pathology, its effects on cardiac structure and function remain poorly characterized. Whether this established MASH model can also produce a progressive and clinically relevant cardiac phenotype remains an important question. Therefore, in this study, we investigated the effects of chronic GAN diet feeding on cardiac function and remodeling in the setting of progressive MASH-like hepatic pathology. Our primary objective was to characterize the cardiac phenotype that develops during prolonged GAN diet exposure and determine whether this established model of MASH also produces features of diet-induced cardiac disease. By integrating longitudinal echocardiography with structural, histological, and molecular analyses of the heart, together with metabolic and hepatic assessments, we aimed to establish a comprehensive baseline characterization of the cardiac and hepatic phenotypes associated with chronic metabolic stress.

## 2. Materials and Methods

Six-week-old male C57BL/6J mice were purchased from the Baylor College of Medicine animal core and housed in individually ventilated cages under standard conditions (12 h light/dark cycle, 45–65% humidity, and 21–23 °C), with water provided ad libitum. Standard normal chow was provided by the animal core, and the GAN diet was purchased directly from Research Diets (New Brunswick, NJ, USA). The GAN diet contained 40 kcal% fat, 22% fructose, and 2% cholesterol; its complete composition is provided in Supplementary Table S1. Following acclimation, mice were randomly allocated to either the normal chow (NC) or GAN diet (GD) group. Sample size was determined based on preliminary echocardiographic data obtained from an independent cohort, with left ventricular ejection fraction (EF) designated as the primary outcome for sample-size planning. In the preliminary cohort, EF was 64.22 ± 2.94% in the normal chow group and 56.51 ± 4.40% in the GAN diet group (n = 8/group), corresponding to an estimated Cohen’s *d* of approximately 2.06. Based on a two-sided α = 0.05, approximately five mice/group would provide 80% power, seven mice/group approximately 90% power, and eight mice/group approximately 97% power to detect this effect. Therefore, n = 8 mice/group was selected for the longitudinal study. The same cohort was followed longitudinally throughout the 6-month dietary intervention, while sample sizes for individual downstream analyses varied according to tissue availability and the technical requirements of each assay. No animals were excluded from the study based on predefined experimental exclusion criteria, and all animals enrolled in the study completed the 6-month dietary intervention and the planned longitudinal assessments. Animals were monitored throughout the study for general health, body condition, activity, grooming, food and water intake, and other signs of distress or illness. All experimental procedures were performed in accordance with the National Institutes of Health Guide for the Care and Use of Laboratory Animals and were approved by the Institutional Animal Care and Use Committee at Baylor College of Medicine (Protocol No. AN-6660).

### 2.1. Analysis of Liver Metabolites (Serum Biochemistry)

Blood samples (200–400 µL) collected using a 5 mm lancet from the submandibular (facial) vein of overnight-fasted, conscious mice were kept on ice and centrifuged at 4000 rpm for 15 min to collect stabilized serum. Serum was subjected to the ELISA for liver metabolite measurements including ALT (alanine aminotransferase), AST (aspartate aminotransferase) and ALP (alkaline phosphatase), which were determined using their specific kits following the manufacturer’s protocols (Teco Diagnostics, Anaheim, CA, USA). Cholesterol and triglycerides contents were measured with Infinity total cholesterol and triglyceride kits [17].

### 2.2. Metabolic Phenotyping (CLAMS and MRI)

Whole-body metabolic parameters were measured via a Comprehensive Lab Animal Monitoring System (CLAMS; Columbus Instruments, Columbus, OH, USA) with the help of the Mouse Metabolism & Phenotyping Core at Baylor College of Medicine, Houston, TX. Mice were individually housed in metabolic chambers under controlled temperature and a 12 h light/dark cycle with ad libitum access to food and water. After a 24 h acclimation period, oxygen consumption (VO_2_), carbon dioxide production (VCO_2_), respiratory exchange ratio (RER = VCO_2_/VO_2_), and energy expenditure were constantly logged. Locomotor activity was measured via infrared beam breaks in both the x-axis and y-axis. Water and food intake were also monitored automatically throughout the study period. Energy expenditure was calculated based on indirect calorimetry measurements and all the parameters were analyzed independently for light and dark cycles to account for circadian variation [18].

Whole-body body composition was assessed in non-fasted mice using a Bruker BioSpin MRI PharmaScan 70/16 (7.0 T) system at the Mouse Metabolism & Phenotyping Core at Baylor College of Medicine, Houston, TX. MRI-based measurements were used to determine whole-body fat mass and lean mass. The resulting body composition parameters were analyzed between the normal chow (NC) and GAN diet (GD) groups.

### 2.3. Echocardiography

Echocardiography was performed with the VisualSonics Vevo 3100 24 h after hair removal. Anesthesia was maintained using isoflurane according to standard protocols developed previously [17]. The amount of isoflurane dispensed (2% in 100% oxygen) was adjusted individually for each animal to maintain a similar heart rate. The body temperature was maintained within the physiological range of 36–38 °C throughout the imaging process using a dedicated heating pad. One-dimensional (M-mode) and two-dimensional (B-mode) echocardiographic images of both the short and long axes were recorded for further analysis. Images from both modes were analyzed using VEVO software to measure ejection fraction (EF), fractional shortening (FS), left ventricle interior diameter (LVID), and left ventricle posterior wall thickness.

To assess diastolic function, Doppler flow profiles were obtained with pulsed wave Doppler in the apical four-chamber view. The sample volume was positioned at the level of the mitral leaflet tips within the mitral orifice, aligned parallel to blood flow, to obtain maximal transmitral flow velocities. Aortic outflow profile and mitral inflow were recorded simultaneously to acquire E wave, A wave and IVRT measurements.

The pulsed wave Doppler was placed at the septal corner of the mitral annulus in the apical four-chamber view using tissue Doppler imaging. E’ was determined as peak mitral annular velocity during early filling [19].

### 2.4. Heart and Liver Histology and Immunofluorescence

Heart and liver tissues were collected following terminal euthanasia of the mice according to previously established methods. Half of the liver and heart were snap-frozen for RT PCR, and the rest were subjected to 4% paraformaldehyde for embedding after a PBS wash. Paraffin-embedded sections were prepared at an average thickness of 5–8 μm for Masson’s trichrome, Sirius Red, wheat germ agglutinin (WGA) staining, and immunofluorescence. Masson’s trichrome and Sirius Red staining were performed using commercial staining kits (KTTRBPT and KTPSRPT, respectively; StatLab Medical Products, Lodi, CA, USA) according to the manufacturer’s instructions. For immunostaining, tissues were stained with CD68 and F4/80 antibodies (1:1000 dilution) following our previously published lab protocols [17]. Histopathological features were scored across multiple microscopic fields for each mouse, and the mean score across fields was calculated to obtain a single mouse-level value for each parameter. Mice were considered the experimental unit for statistical analysis [20].

### 2.5. Real-Time Polymerase Chain Reaction and Gene Expression Analysis

Total RNA was isolated from frozen heart samples using Trizol reagent followed by reverse transcription into cDNAs using an iScript cDNA synthesis kit (Bio-Rad, Hercules, CA, USA). The specific pair of primers, prepared using the Universal Probe Library Assay The specific pair of primers prepared using Universal Probe Library Assay Design Center tool from Roche were provided in Supplementary Table S3. Real-time PCR was performed with 185 FastStart Universal Probe Master mix, specific primers and probes for each gene in Roche 186 Lightcycler 480 PCR machine. Reaction mixtures were incubated at 95 °C for 10 min followed by 55 cycles at 95 °C for 10 sec and 60 °C for 30 sec. The 55-cycle amplification protocol was based on our laboratory’s established and standardized RT-qPCR conditions and was applied consistently across all samples. 18S (ThermoFisher Scientific, Waltham, MA, USA) was used as a housekeeping gene.

### 2.6. Western Blotting

For Western blot analysis, cardiac tissues were homogenized in protein lysis buffer with protease inhibitors, and protein concentrations were determined. Equal protein amounts were separated by SDS-PAGE and transferred to PVDF membranes. Membranes were blocked and incubated overnight at 4 °C with ANP (1:2000; Invitrogen, Carlsbad, CA, USA, PA5-29559) or BNP (1:2000; Invitrogen, PA5-96084) antibodies, followed by incubation with HRP-conjugated secondary antibodies. β-actin was detected using an HRP-conjugated antibody (C4; 1:5000; Santa Cruz Biotechnology, Dallas, TX, USA, sc-47778) for 1 h at room temperature. Protein bands were visualized by enhanced chemiluminescence and quantified by densitometry. ANP and BNP levels were normalized to β-actin and expressed as fold change relative to NC.

### 2.7. Statistical Analysis

Data were distributed normally and are shown as mean ± SEM. Differences in the mean were analyzed by one- or two-way ANOVA followed by post hoc analysis using Tukey’s test. Some of the differences were also analyzed using Student’s *t*-test and Fisher’s exact test. All the analyses were performed by GraphPad Prism version 10.0 and *p* ≤ 0.05 was considered statistically significant.

Investigators responsible for animal husbandry and dietary interventions were aware of group allocation during the conduct of the experiment. Outcome assessment and data analysis were performed independently by two investigators who were blinded to group allocation. The analyses were compared and verified to ensure consistency of the results and their interpretation.

## 3. Results

### 3.1. Chronic Dietary Stress Induces Early Cardiac Dysfunction and Cardiomyocyte Hypertrophy

**Figure 1:**
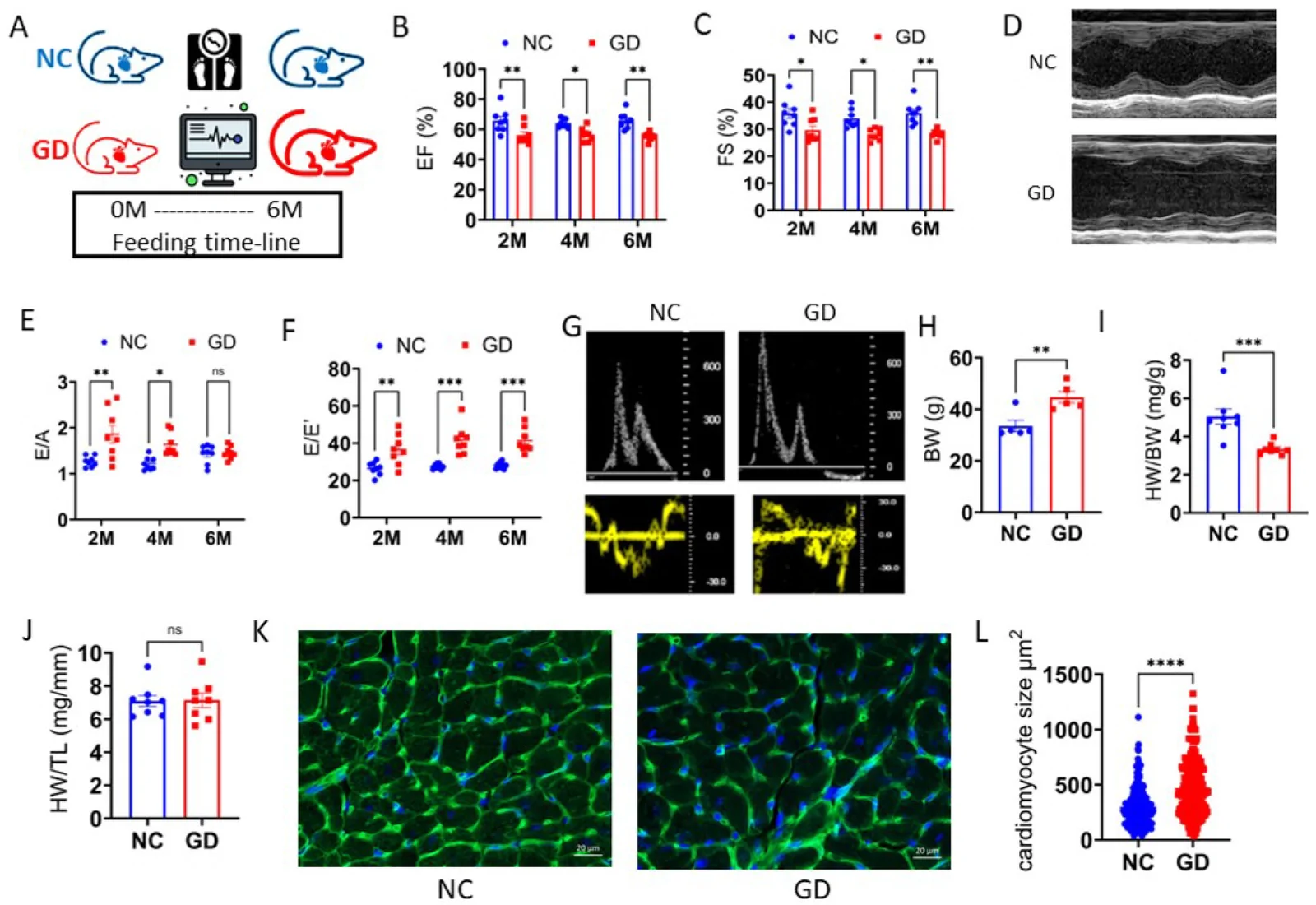
**Chronic dietary stress induces early cardiac dysfunction and cardiomyocyte hypertrophy**. (**A**) Experimental design illustrating two groups of mice fed either normal chow (NC) or the GAN diet (GD) for 6 months. Cardiac function and metabolic parameters were assessed every 2 months. (**B**,**C**) Progressive reductions in ejection fraction (EF) and fractional shortening (FS) in GD-fed mice at 2, 4, and 6 months indicate impaired systolic function. (**D**) Representative longitudinal M-mode echocardiographic images. (**E**,**F**) Increased E/A and E/E′ ratios over time demonstrate the development of diastolic dysfunction in GD-fed mice. (**G**) Representative Doppler images of transmitral inflow and tissue Doppler waveforms obtained from the left ventricular posterior wall, used to assess left ventricular diastolic function and regional wall motion abnormalities, respectively. (**H**) Increased body weight (BW) in GD-fed mice. (**I**) Reduced heart weight normalized to body weight (HW/BW) in GD-fed mice. (**J**) No significant difference in heart weight normalized to tibial length (HW/TL) between groups (n = 8 mice). (**K**) Representative wheat germ agglutinin (WGA) immunofluorescence staining and (**L**) quantitative analysis showing increased cardiomyocyte cross-sectional area in GD-fed mice, indicative of cardiac hypertrophy (n = 6 mice/group; 10 sections per mouse). Scale bar = 20 μm. Data are presented as mean ± SEM. Longitudinal echocardiographic parameters, including EF (**B**), FS (**C**), E/A (**E**), and E/E′ (**F**), were analyzed using two-way repeated-measures ANOVA with diet and time as factors, followed by Sidak’s multiple-comparisons test. Body weight (**H**), heart weight/body weight (**I**), and heart weight/tibial length (**J**) were compared between NC and GD groups using an unpaired two-tailed Student’s *t*-test. Cardiomyocyte size (**L**) was compared between groups using an unpaired two-tailed Student’s *t*-test. *p* < 0.05 was considered statistically significant. * *p* < 0.05, ** *p* < 0.01, *** *p* < 0.001, **** *p* < 0.0001.

To investigate the impact of chronic dietary stress (GAN diet) on cardiac structure and function, 6-week-old mice were subjected to the GAN diet for 6 months, while control animals were maintained on standard normal chow (Figure 1A). Body weight, metabolic parameters and cardiac function were recorded every two months. Echocardiographic measurements showed impaired cardiac function in GAN diet-fed mice over time. In particular, left ventricular systolic function was reduced, as observed by decreased ejection fraction (EF) and fractional shortening (FS) (Figure 1B–D). Additionally, diastolic function parameters were altered, as evidenced by increased E/A and E/E′ ratios, indicating impaired relaxation and elevated filling pressures (Figure 1E–G). Concurrently, these findings suggest the development of both systolic and diastolic dysfunction-induced chronic dietary stress caused by the GAN diet. GAN diet-fed mice displayed a significant increase in body weight compared to controls (Figure 1H and ). Heart weight normalized to body weight (HW/BW) was reduced in GAN diet-fed mice (Figure 1I), probably indicating the marked increase in overall body mass. However, heart weight normalized to tibial length (HW/TL), a representative of body size independent of fat mass, was not altered (Figure 1J), proposing that gross cardiac hypertrophy is not noticeable at this stage.

**Figure 2:**
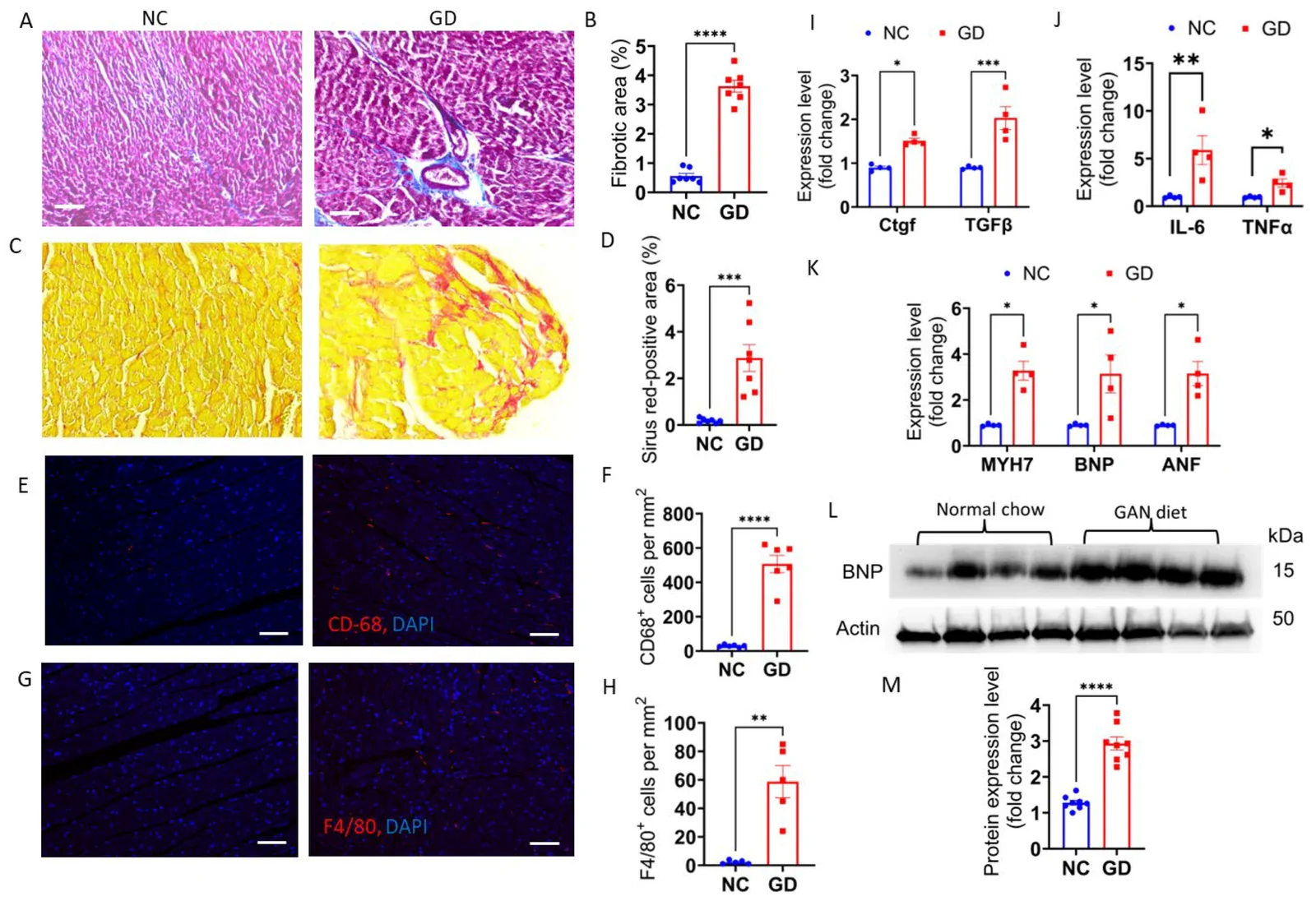
**Chronic dietary stress induces cardiac fibrosis and inflammation.** (**A**) Representative Masson’s trichrome-stained cardiac sections and (**B**) quantitative analysis showing increased myocardial fibrotic area in GD-fed mice compared with NC controls (n = 7 mice/group; eight sections/mouse). (**C**) Representative Sirius Red-stained cardiac sections and (**D**) quantitative analysis showing increased collagen deposition in GD-fed mice compared with NC controls (n = 7 mice/group; four sections/mouse). Scale bars = 200 μm. (**E**,**G**) Representative CD68 and F4/80 immunostaining, respectively, and (**F**,**H**) corresponding quantitative analyses showing increased macrophage infiltration in GD-fed mice (n = 6 mice/group; eight sections/mouse). Scale bars = 20 μm. (**I**) Increased *Ctgf* and *Tgfβ* expression supports activation of profibrotic signaling in GD hearts. (**J**) Increased *Il6* and *Tnfα* expression indicates enhanced cardiac inflammatory signaling in GD-fed mice. (**K**) Increased expression of *Myh7*, *Nppb* (BNP), and *Nppa* (ANF) is consistent with pathological cardiac remodeling in GD-fed mice (n = 4 biologically independent experiments). (**L**) Representative Western blot showing increased BNP protein abundance in cardiac tissue from GD-fed mice compared with NC controls, with actin used as the loading control. (**M**) Quantitative analysis of BNP protein expression normalized to actin, demonstrating increased BNP protein abundance in GD-fed mice compared with NC controls (n = 8 biologically independent experiments). Data are presented as mean ± SEM. Comparisons between NC and GD groups were performed using an unpaired, two-tailed Student’s *t*-test. *p* < 0.05 was considered statistically significant (* *p* < 0.05, ** *p* < 0.01, *** *p* < 0.001, **** *p* < 0.0001).

Consistent with the observed functional impairment, wheat germ agglutinin (WGA) staining revealed increased cardiomyocyte cross-sectional area in GAN diet-fed mice. This finding was further supported by the upregulation of hypertrophic markers, including *Myh7*, *Anf* and *Bnp*, confirming the presence of pathological cardiac remodeling (Figure 1K,L and Figure 2I). Although no significant differences in heart size or left ventricular posterior wall thickness were observed (Supplementary Figure S1A–C), histological and molecular analyses demonstrated cardiomyocyte hypertrophy and remodeling, suggesting that cellular and molecular cardiac alterations may occur before detectable changes in gross cardiac structure.

### 3.2. Chronic Dietary Stress Induces Cardiac Fibrosis and Inflammation

Based on the observed cardiac dysfunction, we next investigated whether chronic GAN diet exposure was associated with pathological cardiac remodeling, characterized by fibrosis, inflammation, and activation of a hypertrophic gene program. Masson’s trichrome staining demonstrated a marked increase in cardiac fibrotic area in GAN diet-fed (GD) mice compared with normal chow (NC) controls (Figure 2A,B). Consistent with these findings, Sirius Red staining revealed increased collagen deposition in GD hearts (Figure 2C,D). To further assess inflammatory remodeling, immunofluorescence staining demonstrated increased numbers of CD68- and F4/80-positive cells in GD hearts, indicating enhanced macrophage accumulation in the myocardium (Figure 2E–H). Consistent with the histological findings, cardiac gene expression analysis showed increased expression of the profibrotic markers *Ctgf* and *Tgfβ* and the inflammatory cytokines *Il6* and *Tnfα* in GD hearts compared with NC controls (Figure 2I,J). In addition, expression of the hypertrophic/remodeling-associated genes *Myh7*, *Nppa*, and *Nppb* was significantly increased in GD hearts (Figure 2K). Increased expression of *Myh7*, *Nppa*, and *Nppb*, together with increased ANP and BNP protein abundance, further supports activation of a pathological cardiac remodeling program in GD-fed mice (Figure 2K–M; Supplementary Figure S2). Collectively, these findings demonstrate that chronic GAN diet exposure is associated with substantial fibrotic and inflammatory remodeling and activation of a pathological cardiac remodeling program in the heart, providing structural and molecular evidence accompanying the systolic and diastolic dysfunction observed in GD-fed mice.

### 3.3. Chronic Dietary Stress Promotes Metabolic Dysfunction, Hypercholesterolemia, and Hepatic Injury

**Figure 3:**
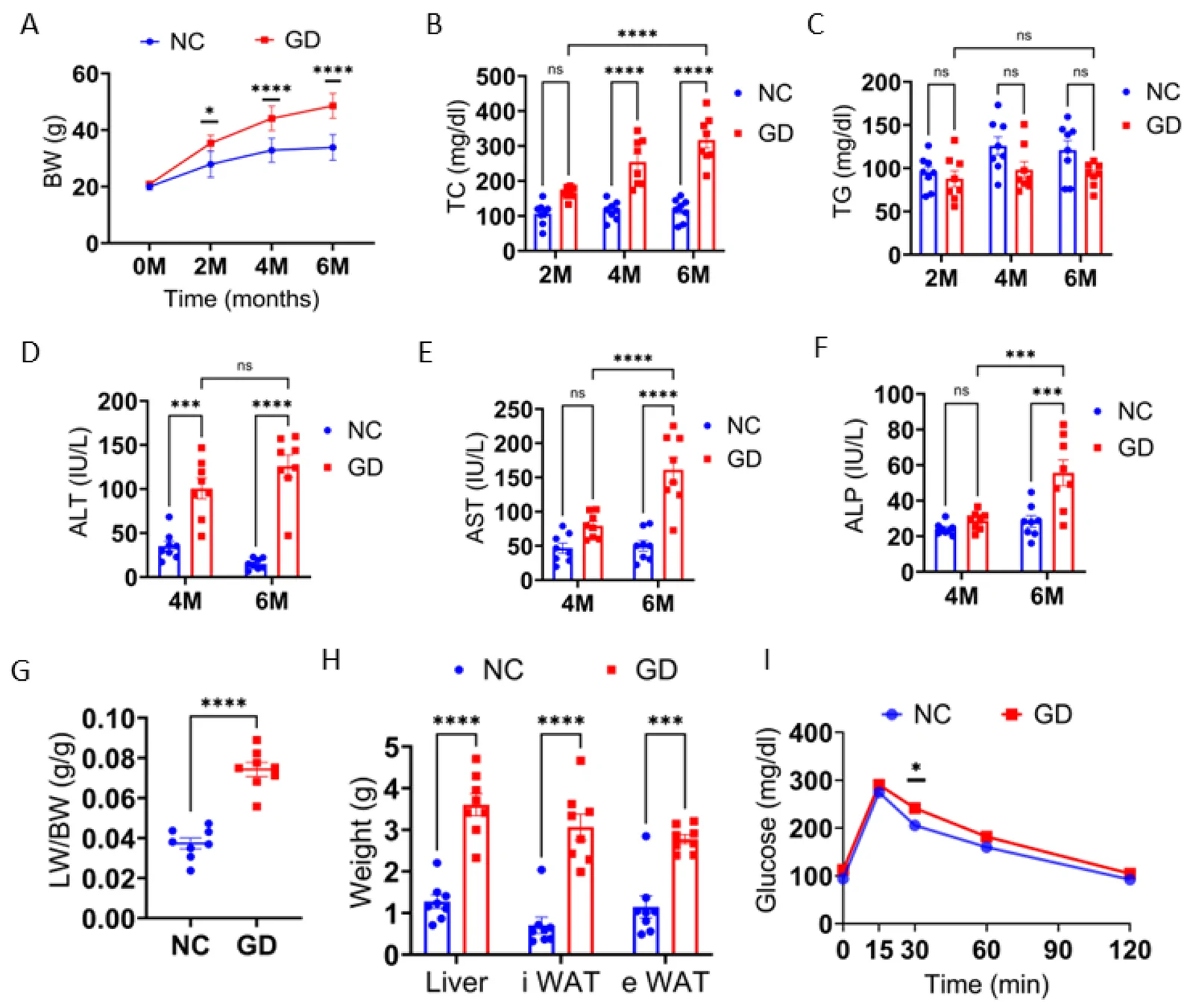
**Chronic dietary stress promotes metabolic dysfunction, hypercholesterolemia, and hepatic injury.** (**A**) Progressive increase in body weight (BW) in GD-fed mice, indicative of sustained metabolic stress. (**B**) Elevated total cholesterol (TC) levels from 2 to 6 months of dietary intervention, demonstrating the development of hypercholesterolemia. (**C**) No significant differences in circulating triglyceride (TG) levels between groups. (**D**–**F**) Increased serum levels of alanine aminotransferase (ALT), aspartate aminotransferase (AST), and alkaline phosphatase (ALP), indicating hepatocellular injury and liver dysfunction in GD-fed mice. (**G**) Increased liver weight normalized to body weight (LW/BW), consistent with hepatic enlargement and injury. (**H**) Increased liver weight as well as inguinal white adipose tissue (iWAT) and epididymal white adipose tissue (eWAT) mass in GD-fed mice, reflecting enhanced adiposity and metabolic remodeling. (**I**) Oral glucose tolerance test (OGTT) demonstrating a transient increase in blood glucose in GD-fed mice at 30 min after glucose administration. Data are presented as mean ± SEM; n = 8 mice in each group. Longitudinal body weight (**A**) and glucose measurements during OGTT (**I**) were analyzed using two-way repeated-measures ANOVA followed by Sidak’s multiple-comparisons test, while TC (**B**), TG (**C**), ALT (**D**), AST (**E**), ALP (**F**), liver weight/body weight (**G**), and liver, inguinal white adipose tissue (iWAT), and epididymal white adipose tissue (eWAT) weights (**H**) were compared using an unpaired two-tailed Student’s *t*-test. *p* < 0.05 was considered statistically significant (* *p* < 0.05, *** *p* < 0.001, **** *p* < 0.0001.; ns, not significant).

Based on the findings from cardiac function, fibrosis, and inflammation, we next examined whether chronic dietary stress induces systemic metabolic alterations and liver injury. GAN diet-fed mice presented a progressive increase in body weight from 2 to 6 months of the feeding period compared to controls (Figure 3A), exhibiting sustained metabolic stress. Moreover, GAN diet-fed mice exhibited elevated circulating cholesterol levels that progressively worsened over time (Figure 3B), whereas triglyceride levels remained unchanged (Figure 3C). During the glucose tolerance test, GD-fed mice exhibited higher blood glucose levels at 30 min compared with NC mice, whereas fasting glucose and glucose levels at later time points were comparable between groups (Figure 3I), indicating a transient alteration in glucose tolerance. All three serum markers of liver injury, including alanine aminotransferase (ALT), aspartate aminotransferase (AST), and alkaline phosphatase (ALP), were significantly elevated in GAN diet-fed mice and progressively worsened over time (Figure 3D–F), indicating hepatocellular damage and biliary stress. GAN diet-fed mice also showed increased liver weight and liver weight to body weight ratio (LW/BW), consistent with hepatic steatosis and liver injury. Furthermore, both epididymal (eWAT) and inguinal (iWAT) fat deposits were markedly increased in GAN diet-fed mice (Figure 3G,H), indicating increased adiposity.

### 3.4. The GAN Diet Induces Metabolic Inflexibility, Increased Adiposity, and Reduced Activity

**Figure 4:**
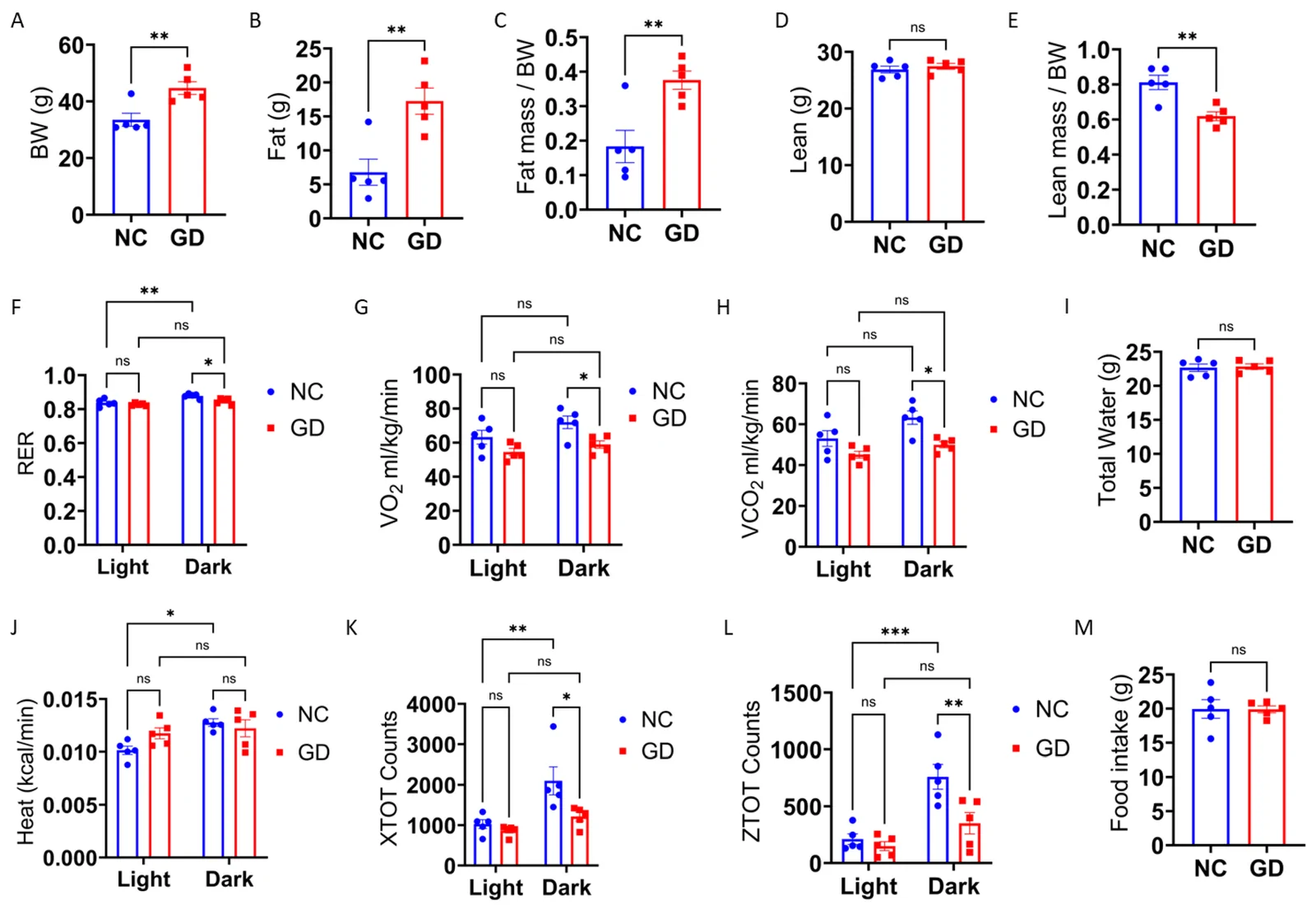
**GAN diet is associated with increased adiposity, altered metabolic parameters, and reduced physical activity.** (**A**–**C**) GD-fed mice exhibited increased body weight, fat mass, and fat mass normalized to body weight (fat mass/BW), indicating increased adiposity. (**D**) No significant difference was observed in lean mass between groups. (**E**) Lean mass normalized to body weight (lean mass/BW) was significantly reduced in GD-fed mice compared with NC controls. (**F**) Respiratory exchange ratio (RER) was lower in GD-fed mice during the dark cycle, consistent with a relative shift toward greater lipid utilization. (**G**,**H**) Oxygen consumption (VO_2_) and carbon dioxide production (VCO_2_) were lower in GD-fed mice during the dark cycle. (**I**) Total water intake was comparable between groups. (**J**) Energy expenditure, measured as heat production (kcal/min), differed between groups during the light cycle but not during the dark cycle. (**K**,**L**) Decreased ambulatory activity (XTOT counts) and total movement (ZTOT counts) indicate reduced locomotor and overall physical activity in GD-fed mice. (**M**) No significant difference in food intake was observed between NC and GD groups. Data are presented as mean ± SEM, n = 5 mice per group. NC and GD groups were compared using an unpaired two-tailed Student’s *t*-test, while light/dark cycle parameters were analyzed using two-way ANOVA with Sidak’s multiple-comparisons test. Because GD mice differed substantially in body composition, VO_2_, VCO_2_, RER, and heat production were also evaluated using ANCOVA with lean mass as a covariate; the corresponding adjusted analyses are provided in Table 1. (* *p* < 0.05, ** *p* < 0.01, *** *p* < 0.001).

To characterize the metabolic adaptations associated with GAN diet feeding, we performed comprehensive metabolic profiling and body composition analyses using CLAMS and MRI, respectively. GAN diet-fed mice showed markedly increased body weight and fat mass compared with mice fed normal chow (NC), resulting in a significantly higher fat mass-to-body weight ratio (fat mass/BW) (Figure 4A–C). In contrast, absolute lean mass did not differ significantly between groups, whereas the lean mass-to-body weight ratio (lean mass/BW) was significantly reduced in GAN diet-fed mice (Figure 4D,E).

Indirect calorimetry revealed a significantly lower respiratory exchange ratio (RER) in GAN diet-fed mice during the dark cycle, consistent with a relative shift toward greater lipid utilization (Figure 4F). Lower oxygen consumption (VO_2_) and carbon dioxide production (VCO_2_) were also observed during the dark cycle in GAN diet-fed mice (Figure 4G,H). Because the substantial differences in body composition could influence interpretation of these parameters, we additionally performed ANCOVA with lean mass as a covariate (Table 1). After adjustment for lean mass, the difference in VO_2_ was no longer significant during the light cycle (*p* = 0.129) but remained lower in GAN diet-fed mice during the dark cycle (*p* = 0.032). Similarly, VCO_2_ did not differ significantly between groups during the light cycle after adjustment (*p* = 0.132) but remained lower in GAN diet-fed mice during the dark cycle (*p* = 0.013). RER showed no significant difference during the light cycle after adjustment (*p* = 0.411), whereas it remained lower in GAN diet-fed mice during the dark cycle (*p* = 0.023).

Energy expenditure, measured as heat production (kcal/min), showed a modest increase in GAN diet-fed mice during the light cycle, with no significant difference observed during the dark cycle (Figure 4J). Consistent with these findings, lean mass-adjusted analysis demonstrated higher heat production in GAN diet-fed mice during the light cycle (*p* = 0.035), whereas no significant difference was observed during the dark cycle (*p* = 0.599). Thus, these data do not support a generalized reduction in energy expenditure associated with GAN diet feeding. In contrast, GAN diet-fed mice exhibited significantly reduced ambulatory activity (XTOT counts) and total movement (ZTOT counts), indicating reduced locomotor and overall physical activity (Figure 4K,L). Water and food intake did not differ significantly between groups (Figure 4I,M).

Collectively, these findings demonstrate that a GAN diet is associated with increased adiposity, altered substrate utilization and metabolic gas exchange, and reduced locomotor activity. The persistence of lower dark-cycle VCO_2_ and RER, together with the lean mass-adjusted analyses, indicates that these metabolic alterations are not fully explained by differences in lean mass. These systemic metabolic alterations accompanied the cardiac and hepatic abnormalities observed in GAN diet-fed mice.

### 3.5. The GAN Diet Promotes an Advanced MASH Phenotype and Fibrotic Remodeling

**Figure 5:**
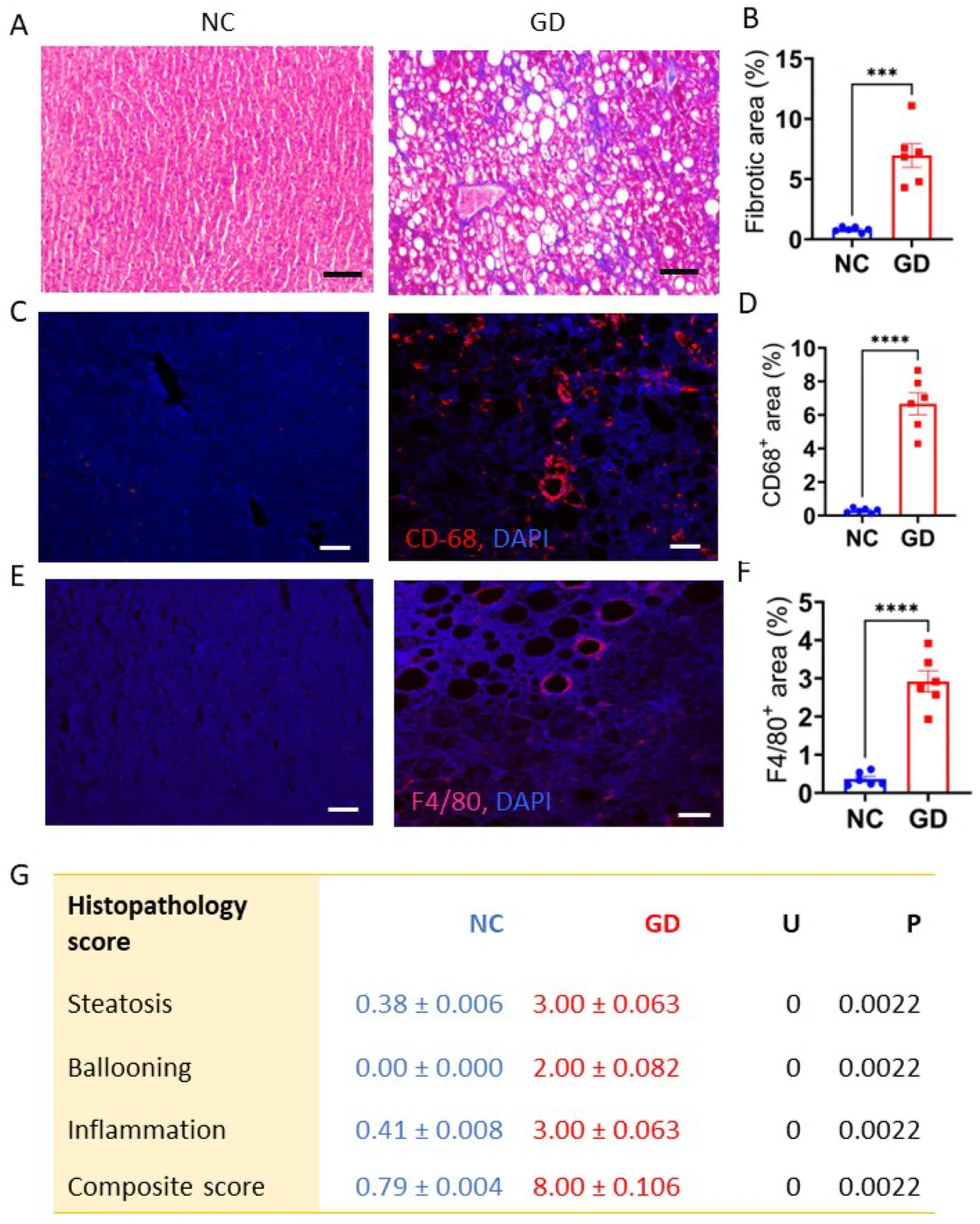
**GAN diet promotes an advanced MASH phenotype and fibrotic remodeling.** (**A**) Representative Masson’s trichrome staining and (**B**) quantitative analysis demonstrating marked hepatic fibrosis in GD-fed mice compared with NC controls (n = 6 mice/group; eight sections per mouse). Scale bar = 100 μm. (**C**–**F**) Representative photomicrographs and quantitative analyses of CD68 and F4/80 immunostaining showing increased macrophage infiltration and hepatic inflammation in GD-fed mice. Scale bar = 20 μm. (**G**) Histopathological assessment of steatosis, hepatocellular ballooning, and lobular inflammation in normal chow NC- and GAN diet GD-fed mice. The NAFLD Activity Score (NAS) was calculated as the sum of the steatosis, ballooning, and inflammation scores. GD-fed mice showed significantly higher steatosis, ballooning, and inflammatory scores, resulting in a higher composite NAS compared with NC-fed mice. Values are presented as mean ± SEM (*n* = 6 mice/group). Individual mouse-level data are provided in Supplementary Table S2. Statistical comparisons were performed using an exact two-sided Mann–Whitney U test; the U statistic was 0 and the exact *p* value was 0.0022 for each comparison. Holm correction for multiple comparisons is provided in Supplementary Table S2. Data are presented as mean ± SEM unless otherwise indicated. All comparisons were performed using an unpaired two-tailed Student’s *t*-test. (*** *p* < 0.001, **** *p* < 0.0001).

To confirm the pathological remodeling and MASH phenotype, we performed histological assessment of the liver tissues. Individual hepatic histopathological scores in normal chow (NC)- and GAN diet (GD)-fed mice are shown in Supplementary Table S2. GAN diet-fed mice showed significantly increased collagen deposition, as assessed by Masson trichrome staining, confirming the increased hepatic fibrosis. This was supplemented by pronounced hepatocellular ballooning and extensive lipid accumulation, consistent with severe steatosis (Figure 5A,B). In addition, increased CD68- and F4/80-positive staining indicated enhanced hepatic macrophage infiltration, suggesting a heightened hepatic inflammation (Figure 5C–F). The NAFLD Activity Score (NAS) was substantially increased in the GAN diet group, driven by significantly higher steatosis, ballooning, and inflammation scores compared with control mice (Figure 5G). Together, these results demonstrated that chronic dietary stress (GAN diet) provokes vigorous hepatic remodeling characterized by steatosis, inflammation, and fibrosis, similar to progressive MASH pathology.

## 4. Discussion

**Figure 6:**
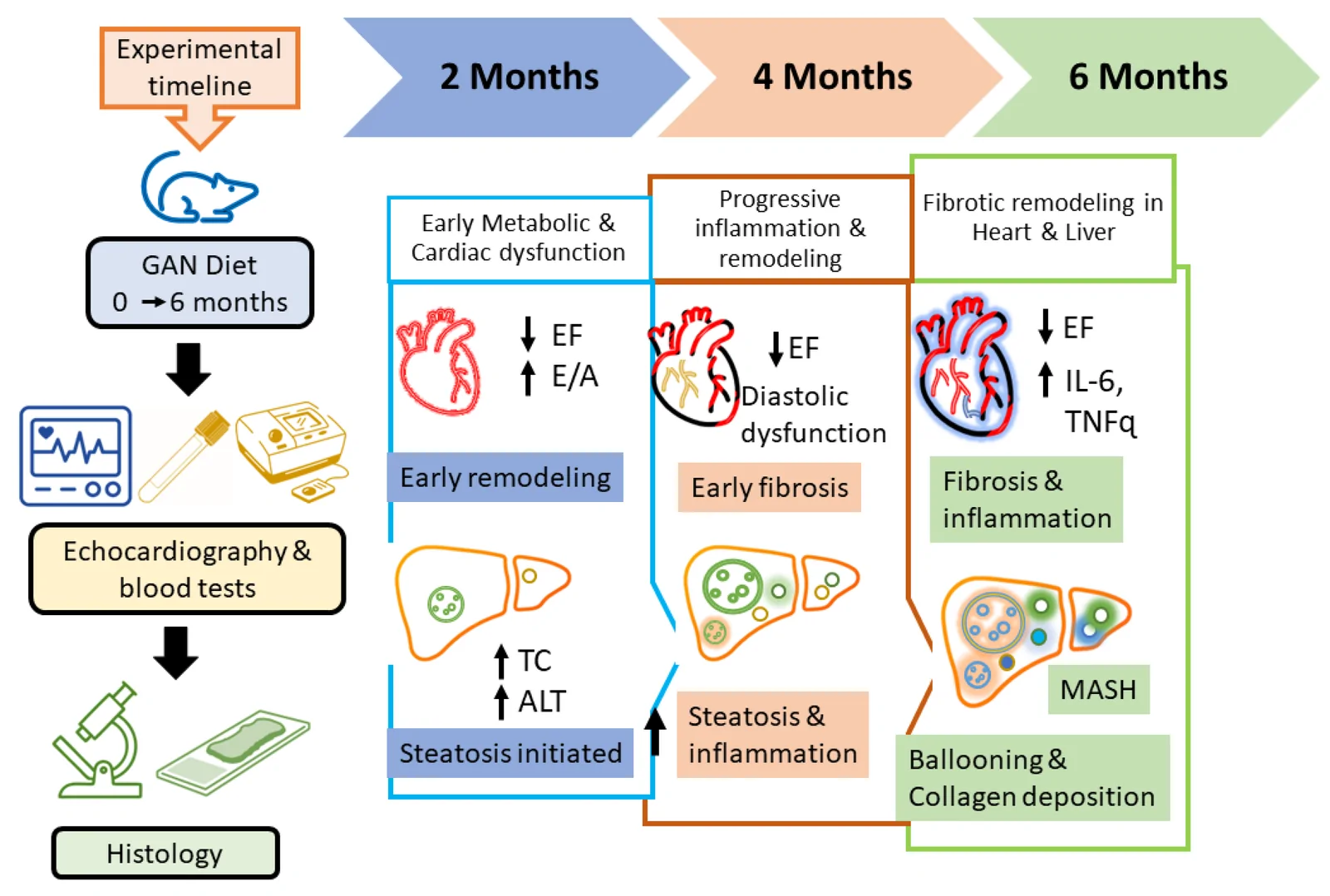
**Experimental design and graphical summary illustrating the progression of chronic dietary stress-induced cardiometabolic dysfunction.** The schematic depicts the experimental timeline and the sequential effects of GAN diet feeding, highlighting the development of obesity, hypercholesterolemia, impaired glucose homeostasis, and systemic inflammation. These metabolic disturbances promote hepatic steatosis, inflammation, fibrosis, and progression to a MASH-like phenotype, while simultaneously driving cardiac inflammation, cardiomyocyte hypertrophy, fibrotic remodeling, and both systolic and diastolic dysfunction. Together, these findings demonstrate how chronic dietary stress induces coordinated pathological changes in the liver and heart, contributing to progressive cardiometabolic disease. Gubra Amylin NASH (GAN); ejection fraction (EF); fractional shortening (FS); total cholesterol (TC); alanine aminotransferase (ALT); metabolic dysfunction-associated steatohepatitis (MASH).

In summary, our findings characterize a progressive cardiac phenotype associated with chronic GAN diet feeding (40 kcal% fat, 22% fructose, and 2% cholesterol) that develops alongside MASH-like hepatic pathology, including steatosis, fibrosis, and inflammation (Figure 6). Chronic metabolic disease involves complex interactions among multiple organs, including the heart and liver; however, although dietary models have been widely used to study MASH, their effects on cardiac biology remain poorly characterized [21]. Several dietary models have previously demonstrated that chronic nutritional stress can induce cardiac abnormalities. For example, prolonged high-fat diet feeding has been reported to cause obesity, metabolic abnormalities, cardiomyocyte hypertrophy, fibrosis, and impaired cardiac function, supporting the use of dietary interventions to model metabolic cardiac disease [13]. Our findings are consistent with these observations, as GAN diet-fed mice developed progressive cardiac dysfunction accompanied by cardiomyocyte hypertrophy and fibrotic remodeling.

However, the cardiac phenotype produced by dietary interventions can vary considerably depending on diet composition and duration. Conventional high-fat or Western-type diets have produced inconsistent or relatively limited cardiac phenotypes, particularly during the early stages of metabolic disease [10,11]. More recently, Ott et al. reported that a high-fat, carbohydrate-free diet induced cardiac metabolic and mitochondrial dysfunction, reduced cardiac mass, and impaired ATP production, while ejection fraction remained preserved [14]. In contrast, our GAN diet-fed mice exhibited an early decline in EF, together with impaired diastolic function, followed by myocardial inflammation and fibrotic remodeling. These differences suggest that both the composition and chronicity of dietary metabolic stress may influence the nature and severity of the resulting cardiac phenotype.

An important observation in the present study is that cardiac dysfunction emerged before overt cardiac hypertrophy. This temporal pattern is consistent with evidence that metabolic and functional alterations can precede structural remodeling during chronic dietary stress. Altered myocardial substrate utilization may affect energy production, calcium handling, and contractile function, potentially contributing to early systolic or diastolic dysfunction before measurable hypertrophic remodeling occurs [22]. Thus, the early reduction in cardiac function observed in GAN-fed mice may represent an initial response to chronic metabolic and inflammatory stress, preceding the structural remodeling observed at later time points. The molecular mechanisms underlying this temporal sequence were not directly examined and warrant further investigation.

Our hepatic findings are also consistent with the established effects of the GAN diet reported by Hansen et al. [16]. Using the same dietary composition of 40 kcal%, 22% fructose, and 2% cholesterol, Hansen et al. demonstrated that prolonged GAN diet feeding in C57BL/6J mice produced obesity, impaired glucose tolerance, and progressive hepatic pathology characterized by steatosis, lobular inflammation, hepatocyte ballooning, and fibrosis [16]. Similarly, our 6-month GAN diet-fed mice developed progressive hepatic steatosis, inflammation, and fibrosis, accompanied by increased liver injury markers. These findings support the reproducibility of the established hepatic effects of the GAN diet. Importantly, while the study by Hansen et al. primarily focused on the hepatic and metabolic consequences of GAN diet feeding, our study extends the characterization of this model to cardiac structure and function. Thus, the present findings provide additional evidence that the GAN diet produces a progressive cardiac phenotype alongside its established MASH-like hepatic phenotype.

In our study, mice were followed for 6 months, with cardiac systolic and diastolic function assessed longitudinally every 2 months. This approach revealed that cardiac dysfunction was detectable relatively early and progressively worsened with continued GAN diet exposure. The reduction in EF and the increases in E/A and E/E′ ratios are consistent with impaired systolic function and altered diastolic filling, respectively, and resemble features associated with early cardiac dysfunction and heart failure [23]. Although heart weight normalized to body weight was reduced, no difference was observed when heart weight was normalized to tibial length, suggesting that the apparent reduction in relative heart weight was largely related to increased overall adiposity rather than true cardiac atrophy [24]. In contrast, WGA staining demonstrated an increased cardiomyocyte cross-sectional area, indicating hypertrophic remodeling, which was further supported by increased expression of *Myh7, Nppa* (*Anf*), and *Nppb* (*Bnp*) [17]. In addition, increased myocardial inflammation and fibrosis provide further evidence of progressive pathological cardiac remodeling following chronic GAN diet exposure.

An important feature of the GAN model is that these cardiac abnormalities developed in parallel with a progressive MASH-like hepatic phenotype. Thus, the potential value of this model is not that the GAN diet represents the first dietary intervention capable of producing cardiac abnormalities, but rather that it provides an experimental system in which cardiac dysfunction and pathological remodeling can be studied in the context of chronic metabolic stress and established MASH-like liver disease. This integrated phenotype may be particularly useful for future studies investigating the mechanisms underlying cardiometabolic disease and for evaluating therapeutic interventions targeting metabolic, inflammatory, or fibrotic pathways.

This cardiac phenotype developed in the context of profound systemic metabolic dysfunction characterized by increased adiposity, hypercholesterolemia, and dyslipidemia. Indirect calorimetry revealed lower RER, VO_2_, and VCO_2_ during the active dark phase, with the reductions in VCO_2_ and RER remaining significant after adjustment for lean mass. These findings are consistent with altered substrate utilization and metabolic gas exchange rather than a generalized reduction in energy expenditure. Indeed, lean mass-adjusted heat production was higher during the light cycle and was not significantly different during the dark cycle, arguing against a global reduction in energy expenditure. In parallel, GAN diet-fed mice exhibited reduced ambulatory and total locomotor activity, indicating diminished physical activity [25]. Importantly, increased adiposity and reduced locomotion may themselves contribute to or exacerbate cardiac dysfunction, and our study cannot distinguish their independent effects from those of the GAN diet. Cardiac dysfunction was also accompanied by severe hepatic pathology compatible with MASH, including steatosis, inflammation, and fibrosis [26]. The concurrent development of cardiac and hepatic pathology suggests that multiple metabolic and inflammatory factors may contribute to the observed phenotype, potentially including systemic inflammation, lipotoxicity, and profibrotic signaling. However, these mechanisms were not directly assessed in the present study. These findings highlight the multifactorial nature of diet-induced cardiometabolic disease and support the potential utility of this model for investigating the effects of chronic metabolic stress on cardiac and hepatic function.

Despite the strengths of this study, future pharmacologic and genetic intervention studies will be required to provide deeper mechanistic insights into the pathways linking myocardial remodeling to hepatic injury and to determine whether inflammatory, fibrotic, and lipid-handling pathways directly contribute to these phenotypes. Targeted investigation of these pathways may further identify potential therapeutic targets for mitigating both cardiac and hepatic injury. Although this study was conducted in a murine model, the GAN diet recapitulates key features of human metabolic syndrome, MASH, and associated cardiovascular dysfunction, supporting its translational relevance for investigating diet-induced cardiometabolic disease. An additional limitation is that complete blinding during echocardiographic data acquisition was not feasible, because the diet groups developed visibly different body sizes and body compositions. Although echocardiographic image analysis was performed with investigators blinded to group allocation, the operator performing image acquisition could potentially recognize group assignment based on the animals’ gross appearance. Thus, the possibility of subtle observer-related effects during image acquisition cannot be completely excluded. Future studies incorporating blinded acquisition or standardized automated imaging protocols may further minimize this potential source of bias. Given known sex-dependent differences in MASH progression and cardiovascular remodeling, the metabolic, hepatic, and cardiac responses to GAN diet feeding observed in this study may not be generalizable to female mice. Future studies incorporating both sexes will be important to determine whether these phenotypes differ according to sex.

## 5. Conclusions

In conclusion, our findings demonstrate that chronic GAN diet feeding is associated with progressive cardiac dysfunction and pathological remodeling, which occur alongside MASH-like hepatic disease. The accompanying inflammatory and fibrotic changes suggest a potential relationship between systemic metabolic stress, hepatic injury, and cardiac remodeling; however, the observational nature of the present study does not establish a causal role for these pathways. This model therefore provides a valuable platform for further investigating the metabolic, inflammatory, and fibrotic processes associated with diet-induced cardiometabolic disease and for testing their potential causal contributions through targeted interventional studies.

## Figures and Tables

**Table 1:** Lean mass-adjusted analysis of indirect calorimetry parameters in NC and GD mice using ANCOVA.

Outcome	Cycle	Covariate	*p* Value	Direction
VO_2_	Light	Lean mass	0.129	GD lower
VO_2_	Dark	Lean mass	0.032	GD lower
VCO_2_	Light	Lean mass	0.132	GD lower
VCO_2_	Dark	Lean mass	0.013	GD lower
RER	Light	Lean mass	0.411	GD lower
RER	Dark	Lean mass	0.023	GD lower
Heat production	Light	Lean mass	0.035	GD higher
Heat production	Dark	Lean mass	0.599	No significant difference

Data were analyzed by analysis of covariance (ANCOVA), with dietary group (NC vs. GD) as the main factor and lean mass as a covariate. Analyses were performed separately for the light and dark cycles. *p* < 0.05 was considered statistically significant. NC, normal chow; GD, GAN diet; VO_2_, oxygen consumption; VCO_2_, carbon dioxide production; RER, respiratory exchange ratio.

## Data Availability

All data generated or analyzed during this study are included in this published article and its Supplementary Materials. Additional data are available from the corresponding author upon reasonable request.

## Supplementary Materials

The following supporting information can be downloaded at https://www.mdpi.com/article/10.3390/nu18183084/s1, Figure S1: (A–C) No significant differences were observed in heart weight (HW), left ventricular posterior wall thickness at diastole (LVPW;d), or left ventricular internal diameter at diastole (LVID;d) between NC- and GD-fed mice. Data are presented as mean ± SEM; Figure S2: Western blot analysis of ANP protein expression in cardiac tissue. Representative Western blot showing ANP protein expression in cardiac tissue from normal chow (NC)- and GAN diet (GD)-fed mice, with β-actin used as the loading control. ANP protein abundance was increased in GD-fed mice compared with NC controls. (n = 8 biologically independent experiments). Table S1: Composition and nutritional characteristics of the GAN diet and normal chow; Table S2: Individual hepatic histopathological scores in normal chow (NC)- and GAN diet (GD)-fed mice; Table S3: Sequence information for qPCR primers.
