# Supplementary material for: Chronic Dietary Stress Is Associated with Early Cardiac Dysfunction and Progressive MASH Accompanied by Systemic Inflammation and Fibrosis
Source: Nutrients. 2026 Sep 20;18(18):3084. doi: 10.3390/nu18183084 (PMC13610571; doi:10.3390/nu18183084)
Supplement: Supplementary file 1 [file nutrients-18-03084-s001.zip › nutrients-4452412-supplementary.pdf]

Sup. Table S1: Composition and nutritional characteristics of the GAN diet and normal chow

Component	GAN diet (GD)	Laboratory Rodent Diet 5001 (NC)
**Diet type**	Gubra Amylin NASH (GAN) diet	Laboratory Rodent Diet 5001
**Protein sources**	Casein (200 g); L-cystine (3 g)	Ground corn; dehulled soybean meal; fish meal; dried plain beet pulp; dried alfalfa meal; brewers dried yeast; cane molasses; wheat germ; wheat middlings; DL-methionine
**Carbohydrate sources**	Fructose (200 g); sucrose (100 g); Lodex 10 (100 g)	Ground corn; cane molasses; wheat germ; wheat middlings and other grain-derived ingredients
**Fiber source**	Solka Floc, FCC200 (50 g)	Dried plain beet pulp; dehydrated alfalfa meal; grain-derived ingredients
**Fat sources**	Palm oil (135 g); soybean oil (25 g); lard (20 g)	Fat provided by grain, soybean meal, fish meal, and other ingredients; formulation contains not separately listed high-fat oil source in the provided ingredient list
**Cholesterol**	Cholesterol, NF (18 g; **2%**)	Not specifically listed
**Fructose**	22%	Not specifically listed
**Total fat**	40 kcal% of energy	Crude fat ≥4.5%
**Protein**	203 g	Crude protein ≥23.0%
**Fiber**	50 g	Crude fiber ≤6.0%
**Moisture**	—	≤12.0%
**Ash**	—	≤8.0%
**Vitamin/mineral supplementation**	Vitamin mix (V10001C, 1 g); mineral mix (S10026B, 50 g); choline bitartrate (2 g)	Vitamin and mineral supplements including vitamins A, D3, E, K, B vitamins, calcium, phosphorus, zinc, iron, manganese, copper, selenium, iodine, cobalt, etc.
**Other components**	Blue FD&C #1 (0.03 g); Red FD&C #40 (0.03 g)	Calcium carbonate; salt; DL-methionine; choline chloride; other micronutrient supplements
**Primary metabolic characteristics**	High-fat, high-fructose, cholesterol-containing diet designed to induce MASH/NASH-like metabolic and hepatic pathology	Standard complete life-cycle laboratory rodent diet designed for maintenance and long-term biomedical research
**Total formulation shown**	904.05 g	Manufacturer-reported formulation; ingredient percentages not provided in the available product information

Sup. Table S2: Individual hepatic histopathological scores in normal chow (NC) and GAN diet (GD) fed mice.

**Group**	**Mouse**	**Steatosis score**	**Ballooning score**	**Inflammation score**	**Composite histopathology score**
NC	1	0.39	0.00	0.41	0.80
NC	2	0.40	0.00	0.38	0.78
NC	3	0.36	0.00	0.43	0.79
NC	4	0.38	0.00	0.39	0.77
NC	5	0.37	0.00	0.42	0.79
NC	6	0.38	0.00	0.40	0.78
NC	Mean ± SEM	0.380 ± 0.006	0.000 ± 0.000	0.405 ± 0.008	0.785 ± 0.004
GD	1	2.80	2.10	3.10	8.00
GD	2	3.20	1.80	2.80	7.80
GD	3	3.10	1.70	2.90	7.70
GD	4	2.90	2.20	3.10	8.20
GD	5	2.90	2.10	2.90	7.90
GD	6	3.10	2.10	3.20	8.40
GD	Mean ± SEM	3.000 ± 0.063	2.000 ± 0.082	3.000 ± 0.063	8.000 ± 0.106
**U**		0	0	0	0
P (Holm-adjusted)		0.0087	0.0087	0.0087	0.0087
P(raw)		0.0022	0.0022	0.0022	0.0022

Scores represent quantitative histopathological assessments for each mouse. The composite histopathology score was calculated as the sum of the steatosis, ballooning, and inflammation scores and is provided as a descriptive composite measure. Each mouse was considered one biological replicate. Values are mean ± SEM; n = 6 mice/group. Exact two-sided Mann-Whitney U test. Holm correction was applied for the four histopathological comparisons. NC, normal chow; GD, GAN diet.

Sup. Table S3: Sequence information for qPCR primers

**Gene (mouse)**	**Forward primer**	**Reverse primer**
Anf	5'- cacagatctgatggatttcaaga-3'	5'- cctcatcttctaccggcatc-3'
Bnp	5'- gtcagtcgtttgggctgtaac-3'	5'- agacccaggcagagtcagaa-3'
Ctgf	5'- tgacctggaggaaaacattaaga-3'	5'- agccctgtatgtcttcacactg-3'
Myh7	5'- ctcagagctcaagcgggata-3'	5'- aaccagccatctcctctgtc-3'
Tgf-β	5'- tggagcaacatgtggaactc-3'	5'- gtcagcagccggttacca-3'
**(rat)**		
Il-6	5'-cagaggataccacccacaac-3'	5'-ccagtttggaagcatccatc-3'
Tnf-α	5'-tgaacttcggggtgatcg-3'	5'-gggcttgtcactcgagtttt-3'

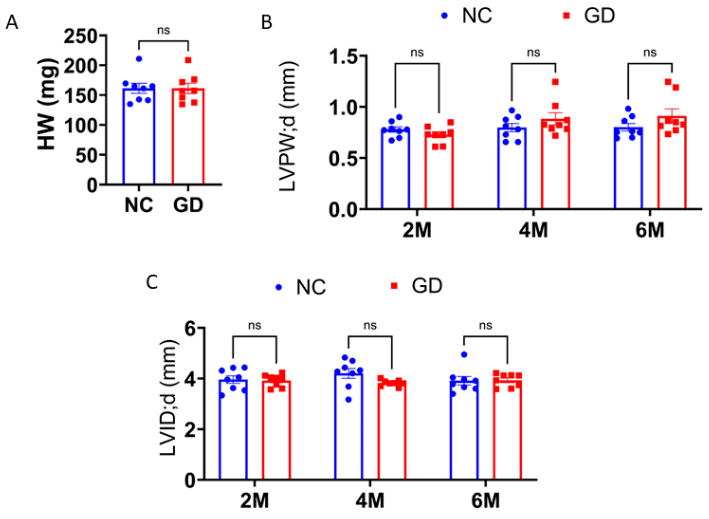

Sup. Figure S1: (A–C) No significant differences were observed in heart weight (HW), left ventricular posterior wall thickness at diastole (LVPW;d), or left ventricular internal diameter at diastole (LVID;d) between NC- and GD-fed mice. Data are presented as mean ± SEM.

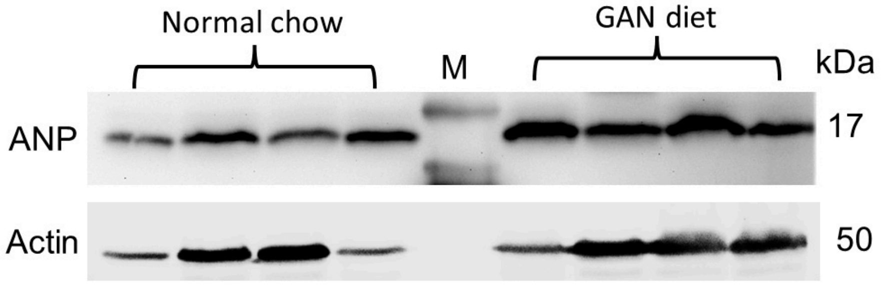

Sup. Figure S2. Western blot analysis of ANP protein expression in cardiac tissue. Representative Western blot showing ANP protein expression in cardiac tissue from normal chow (NC) and GAN diet (GD)-fed mice, with β-actin used as the loading control. ANP protein abundance was increased in GD-fed mice compared with NC controls. (n = 8 biologically independent experiments).
